# Dengue Virus Infection Causes the Activation of Distinct NF-*κ*B Pathways for Inducible Nitric Oxide Synthase and TNF-*α* Expression in RAW264.7 Cells

**DOI:** 10.1155/2015/274025

**Published:** 2015-06-14

**Authors:** Yi-Lin Cheng, Yee-Shin Lin, Chia-Ling Chen, Shu-Wen Wan, Yi-Dan Ou, Chia-Yi Yu, Tsung-Ting Tsai, Po-Chun Tseng, Chiou-Feng Lin

**Affiliations:** ^1^Institute of Basic Medical Science, College of Medicine, National Cheng Kung University, Tainan 701, Taiwan; ^2^Department of Microbiology and Immunology, College of Medicine, National Cheng Kung University, Tainan 701, Taiwan; ^3^Center of Infectious Diseases and Signaling Research, National Cheng Kung University, Tainan 701, Taiwan; ^4^Translational Research Center, Taipei Medical University, Taipei 110, Taiwan; ^5^Department of Microbiology and Immunology, College of Medicine, Taipei Medical University, Taipei 110, Taiwan; ^6^Institute of Clinical Medicine, College of Medicine, National Cheng Kung University, Tainan 701, Taiwan; ^7^Graduate Institute of Medical Sciences, College of Medicine, Taipei Medical University, Taipei 110, Taiwan

## Abstract

Infection with dengue virus (DENV) causes an increase in proinflammatory responses, such as nitric oxide (NO) generation and TNF-*α* expression; however, the molecular mechanism underlying this inflammatory activation remains undefined, although the activation of the transcription factor NF-*κ*B is generally involved. In addition to TNF-*α* production in DENV-infected murine macrophage RAW264.7 cells, inducible NO synthase was transcriptionally and posttranslationally elevated and accompanied by NO generation. NF-*κ*B is known to be activated by DENV infection. Pharmacologically inhibiting NF-*κ*B activation abolishes iNOS/NO biosynthesis and TNF-*α* production. With inhibition, the potential role of NF-*κ*B in oxidative signaling regulation was prevented during DENV infection. Heat-inactivated DENV failed to cause the identified inflammatory responses. Pharmacological inhibition of TLR3 partly decreased NF-*κ*B activation; however, it effectively abolished inducible iNOS/NO biosynthesis but did not inhibit TNF-*α* production. In contrast to TLR3, viral protein NS2B3 also independently contributed to NF-*κ*B activation to regulate TNF-*α* production. These results show the distinct pathways for NF-*κ*B activation caused by DENV infection individually for the regulation of iNOS/NO and TNF-*α* expression.

## 1. Introduction

Dengue virus (DENV), which has 4 serotypes, is a mosquito-borne, enveloped, positive-stranded RNA virus in the Flaviviridae family [1]. It is estimated that approximately 390 million dengue infections occur per year, of which 96 million manifest apparent symptoms [2]. Although most cases of DENV infection are asymptomatic, a variable spectrum of symptoms can occur, ranging from a mild fever to fatal severe dengue hemorrhagic fever (DHF) and dengue shock syndrome (DSS). In DHF/DSS cases, potentially lethal complications, including plasma leakage, severe hemorrhages, and organ failure, can develop [1]. Unfortunately, there is currently no effective vaccine or specific antiviral treatment.

Although the mechanisms of DHF/DSS have not been fully clarified, it is believed that several proinflammatory mediators are involved in hemorrhage development, such as nitric oxide (NO) and tumor necrosis factor-*α* (TNF-*α*) [3, 4]. In monocytes collected from DENV patients with acute fever, inducible NO synthase (iNOS), the major enzyme catalyzing NO production, is significantly increased [5]. As seen in our previous studies, NO can cause endothelial cell damage resulting in vascular leakage, which is induced by antibodies against DENV nonstructural protein (NS) 1 [6–8]. In addition, Yen et al. also observed increased macrophage infiltration and TNF-*α* production in the endothelium of the hemorrhagic tissue and endothelial cell apoptosis in the tissues with enhanced expression of iNOS and nitrotyrosine in DENV-infected mice [9]. Furthermore, TNF-*α* has been reported to induce hemorrhage by causing endothelial cell death, which is associated with increased endothelial cell permeability, in an experimental dengue hemorrhage mouse model [9, 10]. TNF receptor levels have also shown a highly positive correlation with the severity of DHF in patients [11]. However, the underlying molecular mechanism of DENV-induced inflammatory activation remains largely unknown.

Nuclear factor-*κ*B (NF-*κ*B), which is a well-known transcription factor, participates in the regulation of proinflammatory mediators, including iNOS and TNF-*α*. NF-*κ*B is a heterodimer consisting of p65 and p50 subunits, which associate with the inhibitor of NF-*κ*B (I*κ*B) family of inhibitory proteins. Upon I*κ*B degradation, NF-*κ*B translocates to the nucleus and drives the expression of downstream target genes [12]. NF-*κ*B can be activated by several different signaling pathways, including the receptor-mediated signaling of pattern recognition receptors, such as Toll-like receptors (TLRs), retinoic-acid-inducible gene I, melanoma differentiation-associated gene 5 [13], and reactive oxygen species- (ROS-) induced pathways [14]. In DENV infection, Tsai et al. showed that TLR3 plays a major role in interleukin- (IL-) 8 production and viral replication, compared with other TLRs [15]. NF-*κ*B activation during DENV infection has been reported since the 1990s, when several studies showed that DENV can induce NF-*κ*B activation, leading to the induction of apoptosis in different cell types [16–18]; however, the underlying molecular mechanism remains undefined. Recently, Lin et al. clarified that the activation of NF-*κ*B, which leads to endothelium cell apoptosis and hemorrhage development, occurs through the interaction of the DENV NS2B-NS3 (NS2B3) protease complex with I*κ*B*α*/*β*. NS2B3 cleaves I*κ*B*α*/*β* and activates I*κ*B kinase [19]. However, whether NF-*κ*B is involved in DENV-induced iNOS/NO and TNF-*α* production and which pathway mediates these events are still a mystery. Because monocytic cells are the DENV target cells, we used murine mononuclear phagocyte RAW264.7 cells as a model [20, 21]. In this study, we aimed to investigate the role of NF-*κ*B in DENV-induced iNOS/NO and TNF-*α* production and to unveil the underlying molecular mechanism of NF-*κ*B activation.

## 2. Materials and Methods

### 2.1. Antibodies and Reagents

The reagents and antibodies used were translation inhibitor cycloheximide, transcription inhibitor actinomycin D, NF-*κ*B inhibitor caffeic acid phenethyl ester (CAPE), antioxidant N-acetyl-cysteine (NAC), Poly(I:C), TLR3 inhibitor, dimethyl sulfoxide (DMSO), and mouse monoclonal antibody (Ab) specific for *β*-actin (Sigma-Aldrich, St. Louis, MO); Abs against DENV NS1 and envelope (E) (GeneTex, San Antonio, TX); Abs against iNOS and NF-*κ*B (Cell Signaling Technology, Beverly, MA); Abs against isotype control IgG (Millipore, Billerica, MA); donkey anti-goat IgG conjugated with horseradish peroxidase (HRP) (Santa Cruz Biotechnology, Santa Cruz, CA) and goat anti-rabbit IgG conjugated with HRP (Chemicon International, Temecula, CA); rabbit anti-mouse IgG conjugated with HRP (Abcam, Cambridge, MA); and Alexa Fluor 488- and Alexa Fluor 594-conjugated goat anti-mouse and goat anti-rabbit (Invitrogen, Carlsbad, CA). Inhibitors were then dissolved in DMSO prior to dilution with sterile phosphate-buffered saline (PBS) for use in experiments. Other chemical reagents were obtained from Sigma-Aldrich. All drug treatments on cells were assessed for cytotoxic effects using cytotoxicity assays prior to experiments. Noncytotoxic dosages were used in this study.

### 2.2. Cell Culture and Virus Culture

RAW264.7 mouse macrophage was purchased from American Type Culture Collection (ATCC number TIB-71) and were grown on plastic in Dulbecco's modified Eagle's medium (DMEM) with 10% fetal bovine serum (FBS) (Sigma-Aldrich, St. Louis, MO), 100 units of penicillin, and 100 *μ*g/mL of streptomycin at 37°C under 5% CO_2_. Cells were used at 3 to 5 passages. Baby hamster kidney (BHK) cells and C6/36 cells were cultured in DMEM (Invitrogen Life Technologies) containing FBS. DENV2 PL046 strain was maintained in C6/36 cells. Monolayers of C6/36 cells were incubated with DENV at a multiplicity of infection (MOI) of 0.01 and incubated at 28°C in 5% CO_2_ for 5 days. The virus supernatant was further filtered with 0.22 *μ*m filter and then stored at −80°C until use. Virus titer was determined by plaque assay, using the BHK cell line.

### 2.3. DENV Infection

Cells were resuspended at a concentration of 5 × 10^5^ cells/mL in appropriate medium with DENV and incubated for 90 min at 37°C. Then the cells were washed once with DMEM medium, resuspended at a concentration of 5 × 10^5^ cells/mL, and incubated at 37°C with 5% CO_2_. The viral supernatants were checked using plaque assays.

### 2.4. Plaque Assay

BHK-21 cells were plated into 12-well plates (2 × 10^5^ cells/well) and cultured in DMEM under CO_2_-enriched conditions. After adsorption with a serially diluted virus solution for 1 h, the solution was replaced with fresh DMEM containing 2% FBS and 0.5% methyl cellulose (Sigma-Aldrich). Five days after infection, the medium was removed, and the cells were fixed and stained with crystal violet solution containing of 1% crystal violet, 0.64% NaCl, and 2% formalin.

### 2.5. Western Blotting

Harvested cells were lysed in a buffer containing 1% Triton X-100, 50 mM Tris (pH 7.5), 10 mM EDTA, 0.02% NaN_3_, and a protease inhibitor cocktail (Roche Boehringer Mannheim Diagnostics, Mannheim, Germany). After a freeze-thaw cycle, cell lysates were centrifuged at 10,000 ×g at 4°C for 20 min. The lysates were boiled in a sample buffer for 5 min. Proteins were then subjected to SDS-PAGE and transferred to PVDF membranes (Millipore, Billerica, MA, USA) using a semidry electroblotting system. After blocking with 5% skim milk in PBS, the membranes were incubated overnight with a 1 : 1,000 dilution of primary antibodies at 4°C. The membranes were then washed with 0.05% PBS-Tween 20 and incubated with a 1 : 5,000 dilution of HRP-conjugated secondary antibody at room temperature for 1 h. After washing, the membranes were soaked in ECL solution (PerkinElmer Life and Analytical Sciences, Inc., Boston, MA, USA) for 1 min and exposed to an X-ray film (BioMax; Eastman Kodak, Rochester, NY, USA). The relative signal intensity was quantified using ImageJ software (version 1.41o; W. Rasband, National Institutes of Health, Bethesda, MD, USA). The changes in the ratio of proteins compared with the normalized value of untreated cells (indicated protein/*β*-actin) are also determined. One set of representative data obtained from three independent experiments is shown and the data shown as the mean ± SD values from three independent experiments.

### 2.6. TNF-*α* Expression

After treatment, we used a commercial enzyme-linked immunosorbent assay (ELISA) kit (R&D, MN, USA) to detect the concentration of murine TNF-*α* in cell-conditioned culture medium, according to the manufacturer's instructions.

### 2.7. Detection of NO Production

Production of NO was assessed as the accumulation of nitrite (NO_2_^−^) in the medium using a colorimetric reaction with the Griess reagent. Briefly, samples (cell culture supernatants or murine ascites) were mixed with an equal (1 : 1) volume of Griess reagent (0.1%* N*-(1-naphthyl)ethylenediamine dihydrochloride, 1% sulfanilamide, and 2.5% H_3_PO_4_). The absorbance was measured at 540 nm using a 96-well microplate reader (Spectra MAX 340PC, Molecular Devices); data were analyzed using Softmax Pro software. Sodium nitrite was dissolved in double-distilled water and then used as standards (from 1 to 50 *μ*M).

### 2.8. Luciferase Reporter Assay

To analyze NF-*κ*B promoter activity by a luciferase reporter assay, transient transfection was performed using the TurboFect Cell Transfection Reagent (Thermo, PA, USA). In short, cells were cotransfected with 0.2 *μ*g of an NF-*κ*B-promoter-driven firefly luciferase reporter and 0.01 *μ*g of a Renilla luciferase-expressing plasmid (pRL-TK; Promega). Twenty-four hours after the transfection, the cells were infected with DENV for 24 h, lysed, and then harvested for luciferase and* Renilla* measurement, using a luciferase assay system (Dual-Glo; Promega). For each lysate, the firefly luciferase activity was normalized to the* Renilla* luciferase activity to assess transfection efficiencies.

### 2.9. Immunostaining

To detect expression of NF-*κ*B and DENV E protein, cells were fixed with 4% paraformaldehyde, permeabilized with 0.5% Triton X-100, and washed twice with ice-cold PBS. Cells were stained with anti-NF-*κ*B and DENV E Abs and then with Alexa 488-conjugated goat anti-mouse IgG and Alexa 594-conjugated goat anti-rabbit IgG. DAPI (5 *μ*g/mL) was used for nuclear staining. Cells were visualized under a laser-scanning immunofluorescence microscope (IX71; Olympus, PA, USA).

### 2.10. Transfection

Transient transfection was performed using TurboFect Cell Transfection Reagent (Thermo, PA, USA) according to the manufacturer's instructions for optimization and usage. The plasmid expressing Flag-tagged wild-type (WT) NS2B3 and a protease-dead mutant NS2B3 which has a single point mutation changing serine residue 135 of NS3 to alanine (S135A) and its control pCR3.1 were generated previously [22]. After transfection, the cells were cultured for 24 h before the experiments.

### 2.11. Intracellular ROS Assay

Intracellular oxidative stress was measured by dichlorodihydrofluorescein diacetate oxidation. Cells were plated at 1 × 10^5^/well in 96-well plates, cultured overnight, and washed twice with Hank's Buffered Salt Solution (HBSS) before experiments. Cells were exposed to 20 *μ*M 5-(and-6)-chloromethyl-2′,7′-dichlorodihydrofluorescein diacetate, acetyl ester (CM-H_2_DCFDA) (Invitrogen Life Technologies, Carlsbad, CA, USA) for 1 h. Fluorescence was read immediately at wavelengths of 485 nm for excitation and 530 nm for emission on a fluorescence plate reader (Fluoroskan Ascent, Thermo Electron Corporation, Milford, MA, USA).

### 2.12. Statistical Analysis

Data obtained from three independent experiments are presented as the mean ± standard deviation (SD). Statistical analysis of data analyses were performed using Prism version 5 (GraphPad Software, San Diego, CA). Two sets of the data were analyzed by an unpaired Student's *t* test. Three or more sets of data were analyzed by one-way ANOVA with Tukey's multiple-comparison posttest. Statistical significance was set at *P* < 0.05.

## 3. Results

### 3.1. DENV Infection Transcriptionally and Translationally Upregulates iNOS/NO Biosynthesis and TNF-*α* Production

**Figure 1:**
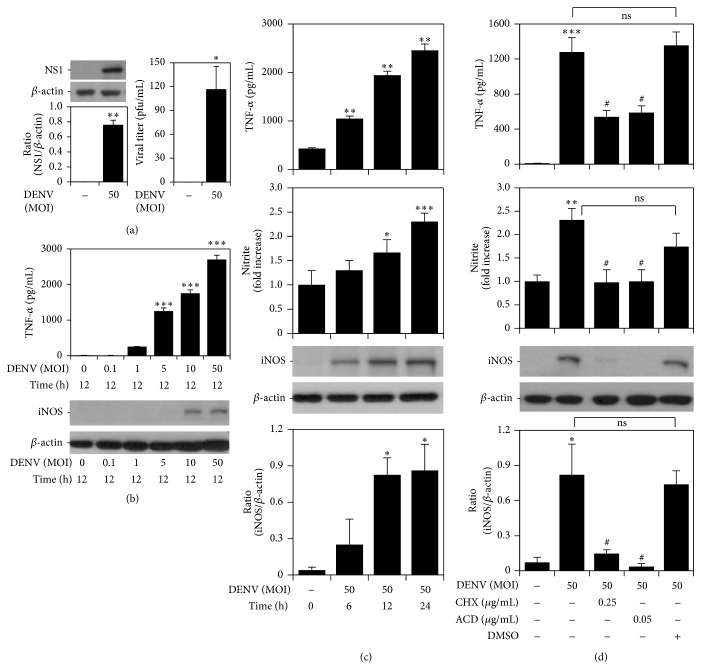
DENV infection induces TNF-*α* production and iNOS/NO biosynthesis through new protein synthesis. (a) RAW264.7 cells infected with DENV serotype 2 PL046 were assessed for DENV NS1 expression by western blot analysis and for viral replication by a plaque assay. ^*∗*^ *P* < 0.05 and ^*∗∗*^ *P* < 0.01, compared with untreated cells. (b, c, and d) ELISA, Griess' reagent, and western blot analysis quantified the expression of TNF-*α* and iNOS/NO in DENV 2-infected cells for the different MOIs, the changes over time, and in the presence of cycloheximide (CHX) and actinomycin D (ACD). DMSO was used as the negative control. ^*∗*^ *P* < 0.05, ^*∗∗*^ *P* < 0.01, and ^*∗∗∗*^ *P* < 0.001, compared with untreated cells. ^#^ *P* < 0.05, compared with DENV. ns: not significant. For western blot results, one set of representative data obtained from three independent experiments is shown. The relative ratio to *β*-actin based on densitometer quantification and analysis using ImageJ software is shown. For all experiments, the quantitative data shown represent mean ± SD values of three independent experiments.

We first examined whether murine mononuclear phagocyte RAW264.7 cells could be efficiently infected by DENV serotype 2 (DENV 2) strain PL046* in vitro*. Using western blotting and plaque assays, we confirmed the expression of viral NS1 and showed that viral titers were significantly increased at 24 h after infection with an MOI of 50 (Figure 1(a)). These results demonstrate that DENV infects RAW264.7 cells* in vitro*. To evaluate the expression of TNF-*α*, NO, and iNOS, we determined the multiple MOIs of DENV infection and the kinetics of response to DENV by ELISA, Griess reagent, and western blotting, respectively. The results showed that a relative increased response of TNF-*α* production and iNOS expression in the multiple MOIs of DENV infection (Figure 1(b)). According to the results, we selected a high MOI of DENV infection for this study and all of these values increased in a time-dependent manner (Figure 1(c)). To determine whether the upregulation of TNF-*α* and iNOS/NO occurred through new protein synthesis, RAW264.7 cells were infected with DENV in the presence or absence of the transcription inhibitor cycloheximide (CHX) and the translation inhibitor actinomycin D (ACD) for 24 h. Treatment with CHX and ACD significantly suppressed the expression of TNF-*α*, NO, and iNOS (Figure 1(d)). These results demonstrate that RAW264.7 cells infected by DENV induce TNF-*α* production and iNOS/NO biosynthesis through new protein synthesis.

### 3.2. DENV Infection Causes NF-*κ*B Activation

**Figure 2:**
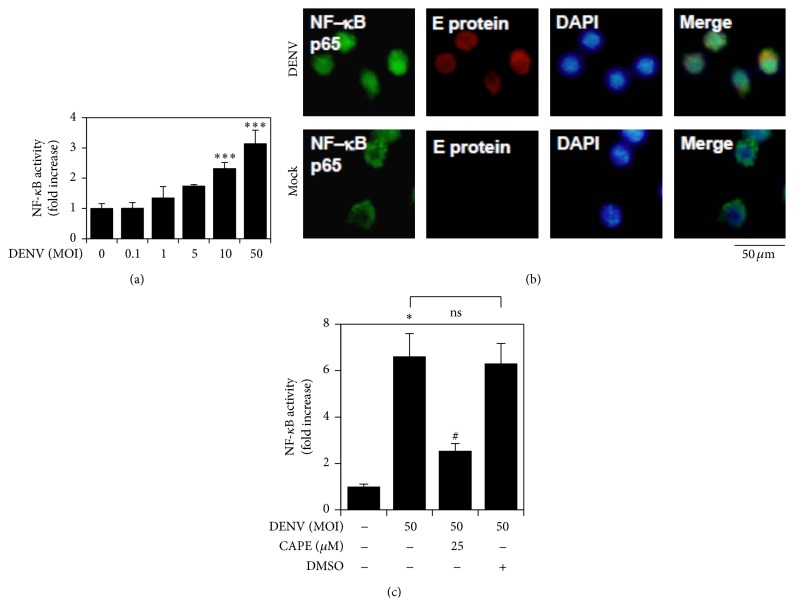
DENV infection promotes NF-*κ*B nuclear translocation and activation. (a) NF-*κ*B reporter assay quantified activation of NF-*κ*B in DENV 2-infected cells for the indicated MOIs 6 h after infection. (b) Immunostaining followed by immunofluorescence microscopy reveals the expression and intracellular localization of the NF-*κ*B p65 (green) and DENV E protein (red) 6 h after infection with DENV 2; DAPI (blue) was used for nuclear staining. Images are representative of three independent experiments. (c) NF-*κ*B reporter assay quantified activation of NF-*κ*B in DENV 2-infected cells pretreated with CAPE. DMSO was used for the negative control. ^*∗*^ *P* < 0.05 and ^*∗∗∗*^ *P* < 0.001, compared with untreated cells. ^#^ *P* < 0.05, compared with DENV. ns: not significant. The quantitative data shown represent the mean ± SD values of three independent experiments.

We next investigated the effects of DENV infection on NF-*κ*B activation. A luciferase reporter assay showed a significant increase of NF-*κ*B activation at the high MOIs of DENV infection (Figure 2(a)). To explore the activation of NF-*κ*B, we used immunocytochemical staining to detect the nuclear translocation of NF-*κ*B in the context of DENV infection. In the infection group, the p65 subunit of NF-*κ*B translocated into the nucleus in E protein-positive cells (Figure 2(b)) at 6 h after infection. Furthermore, a luciferase reporter assay showed that inhibiting NF-*κ*B with CAPE, an inhibitor of NF-*κ*B, blocked DENV-induced NF-*κ*B activation (Figure 2(c)). These results demonstrate that DENV infection activates NF-*κ*B in RAW264.7 cells.

### 3.3. DENV Infection Induces the Expression of TNF-*α*, iNOS, and NO in an NF-*κ*B-Regulated Manner

**Figure 3:**
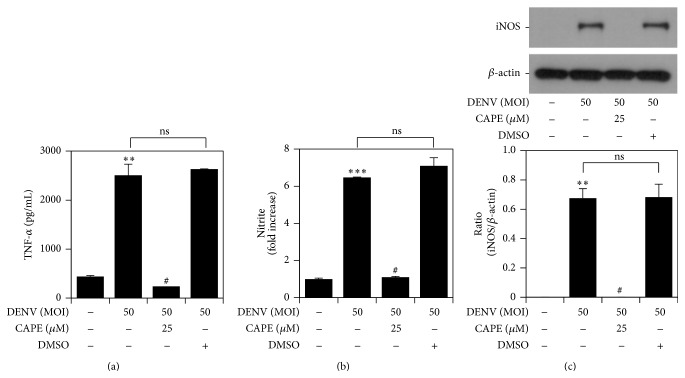
NF-*κ*B activation determines TNF-*α*, NO, and iNOS expression during DENV infection. ELISA (a), Griess' reagent (b), and western blot analysis (c) quantified expression of TNF-*α* and iNOS/NO in DENV 2-infected cells pretreated with CAPE. DMSO was used for the negative control. ^*∗∗*^ *P* < 0.01 and ^*∗∗∗*^ *P* < 0.001, compared with untreated cells. ^#^ *P* < 0.05, compared with DENV. ns: not significant. For western blot results, one set of representative data obtained from three independent experiments is shown. The relative ratio to *β*-actin based on densitometer quantification and analysis using ImageJ software is shown. For all experiments, the quantitative data shown represent the mean ± SD values of three independent experiments.

To further determine the essential role of NF-*κ*B in DENV infection, we examined the effect of NF-*κ*B inhibition on TNF-*α*, NO, and iNOS expression. After treatment with CAPE, DENV-induced TNF-*α* production (Figure 3(a)), NO generation (Figure 3(b)), and iNOS expression (Figure 3(c)) were dramatically downregulated. These results confirmed that NF-*κ*B plays a critical role in DENV-induced TNF-*α*, NO, and iNOS expression.

### 3.4. DENV-Induced ROS Independent NF-*κ*B Activation, TNF-*α* Production, and iNOS/No Biosynthesis

**Figure 4:**
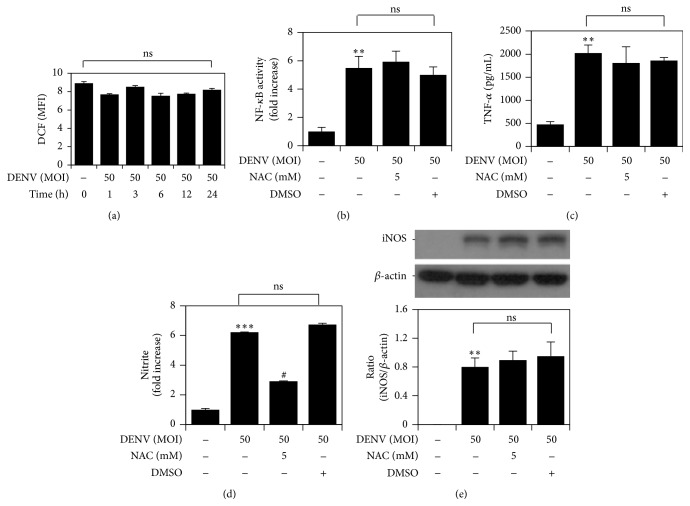
ROS is independent of NF-*κ*B activation followed by TNF-*α* and iNOS expression during DENV infection. (a) CM-H_2_DCFDA-based staining followed by flow cytometric analysis determined ROS generation in RAW264.7 cells infected with DENV 2 at the indicated time points. The mean fluorescence intensity (MFI) of each stain is shown as the mean ± SD of three individual experiments. ns: not significant. In the presence of the ROS-scavenger NAC, NF-*κ*B reporter assay (b), ELISA (c), Griess reagent (d), and western blot analysis (e) quantified the activation of NF-*κ*B and the expression of TNF-*α* and iNOS/NO in DENV 2-infected cells. DMSO was used for the negative control. ^*∗∗*^ *P* < 0.01 and ^*∗∗∗*^ *P* < 0.001, compared with untreated cells. ^#^ *P* < 0.05, compared with DENV. ns: not significant. For western blot results, one set of representative data obtained from three independent experiments is shown. The relative ratio to *β*-actin based on densitometer quantification and analysis using ImageJ software is shown. For all experiments, the quantitative data shown represent the mean ± SD values of three independent experiments.

One of the possible mechanisms modulating NF-*κ*B activation is the ROS-mediated pathway [14]. We examined the possibility of ROS involvement in DENV-induced NF-*κ*B activation followed by TNF-*α*, NO, and iNOS induction. DCFDA staining followed by flow cytometry was used to detect changes in the ROS level in a time-dependent manner. However, there was no significant difference after DENV infection at the indicated time points (Figure 4(a)). Inhibiting ROS using NAC did not affect DENV-induced NF-*κ*B activation (Figure 4(b)). NAC also had no influence on TNF-*α* and iNOS expression (Figures 4(c) and 4(e)). However, inhibition of ROS reduced NO generation significantly during DENV infection (Figure 4(d)). These results indicate that ROS is not responsible for the induction of NF-*κ*B activation followed by TNF-*α* and iNOS upregulation but plays some role in the conversion of iNOS to NO during DENV infection.

### 3.5. Heat-Inactivated DENV Induces NF-*κ*B Activation, TNF-*α* Production, and iNOS/NO Biosynthesis Inefficiently

**Figure 5:**
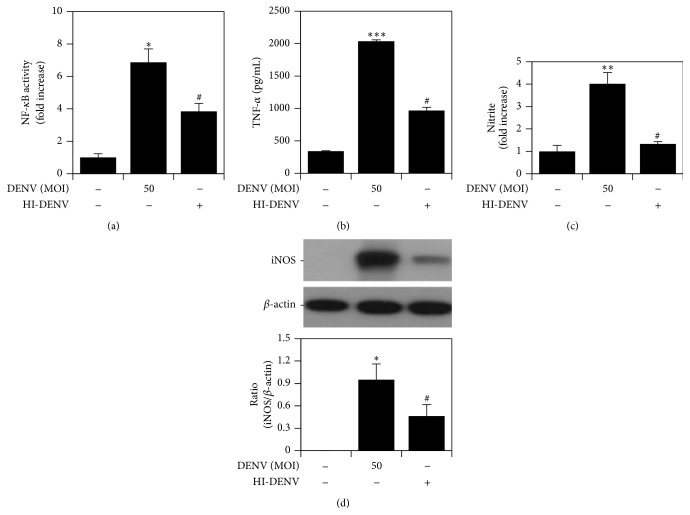
Heat-inactivated DENV does not efficiently cause NF-*κ*B activation, TNF-*α* production, or iNOS/NO biosynthesis. NF-*κ*B reporter assay (a), ELISA (b), Griess' reagent (c), and western blot analysis (d) quantified the activation of NF-*κ*B and the expression of TNF-*α* and iNOS/NO in DENV 2-infected and heat-inactivated DENV 2-treated RAW264.7 cells. ^*∗*^ *P* < 0.05, ^*∗∗*^ *P* < 0.01, and ^*∗∗∗*^ *P* < 0.001, compared with untreated cells. ^#^ *P* < 0.05, compared with DENV. For western blot results, one set of representative data obtained from three independent experiments is shown. The relative ratio to *β*-actin based on densitometer quantification and analysis using ImageJ software is shown. For all experiments, the quantitative data shown represent the mean ± SD values of three independent experiments.

To investigate whether DENV-induced NF-*κ*B activation followed by TNF-*α* production and iNOS/NO biosynthesis occurs in a protein-mediated manner, RAW264.7 cells were inoculated with MOI of 50 of heat-inactivated DENV (HI-DENV). The results revealed that HI-DENV was less efficient at inducing NF-*κ*B activity than live-DENV (Figure 5(a)). Similarly, the levels of TNF-*α* (Figure 5(b)), NO (Figure 5(c)), and iNOS expression (Figure 5(d)) were lower in the HI-DENV group compared with the live DENV group. These experiments show that DENV activates NF-*κ*B followed by TNF-*α* and iNOS/NO biosynthesis through viral proteins.

### 3.6. TLR3 Contributes to NF-*κ*B Activation of iNOS/NO Biosynthesis but Not TNF-*α* Production

**Figure 6:**
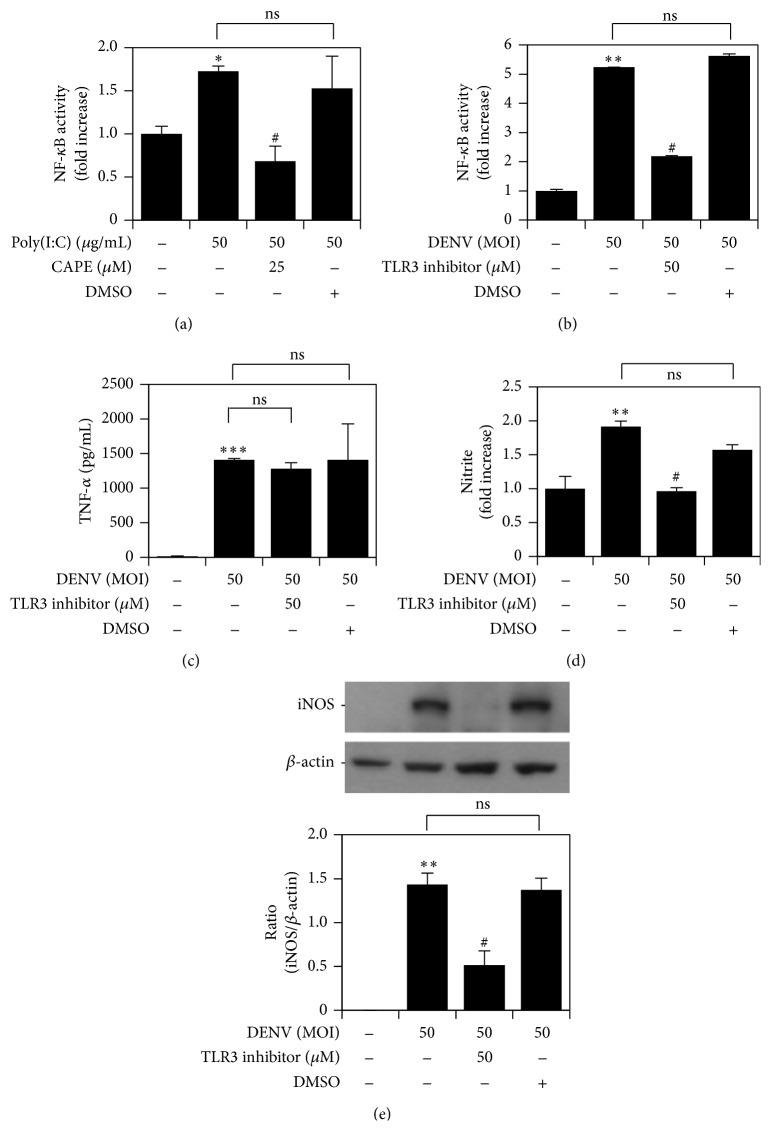
DENV infection induces TLR3-regulated NO and iNOS expression, but not TNF-*α* expression, following NF-*κ*B activation. (a) In the absence and presence of CAPE, an NF-*κ*B reporter assay was performed to measure NF-*κ*B activation in Poly(I:C)-treated RAW264.7 cells. NF-*κ*B reporter assay (b), ELISA (c), Griess reagent (d), and western blot analysis (e) quantified the activation of NF-*κ*B and the expression of TNF-*α* and iNOS/NO in DENV 2-infected RAW264.7 cells pretreated with TLR3 inhibitor. ^*∗*^ *P* < 0.05, ^*∗∗*^ *P* < 0.01, and ^*∗∗∗*^ *P* < 0.001, compared with untreated cells. ^#^ *P* < 0.05, compared with Poly(I:C) or DENV. ns: not significant. For western blot results, one set of representative data obtained from three independent experiments is shown. The relative ratio to *β*-actin based on densitometer quantification and analysis using ImageJ software is shown. For all experiments, the quantitative data shown represent the mean ± SD values of three independent experiments.

According to Tsai et al., TLR3 plays a major role in cellular activation compared with other TLRs during DENV infection [15]. TLR3 is a well-defined receptor for viral RNA, which can induce NF-*κ*B activation during viral infection [13]. We further confirmed the involvement of TLR3 in NF-*κ*B activation and inflammatory response. First, we used the TLR3 agonist polyinosinic-polycytidylic acid poly (Poly(I:C)) to determine whether TLR3 signaling can activate NF-*κ*B. The results revealed that Poly(I:C) activated luciferase activity of NF-*κ*B and that this activity was inhibited by CAPE (Figure 6(a)). Moreover, pharmacological inhibition of TLR3 suppressed NF-*κ*B activation (Figure 6(b)). Surprisingly, there was no difference in TNF-*α* between the presence and absence of TLR3 inhibitor during DENV infection (Figure 6(c)). In contrast, DENV-induced NO (Figure 6(d)) and iNOS (Figure 6(e)) biosynthesis was significantly attenuated by TLR3 inhibition. These results demonstrate that TLR3-activated NF-*κ*B is required for iNOS/NO biosynthesis, but not TNF-*α* expression, during DENV infection.

### 3.7. DENV Protease NS2B3 also Promotes NF-*κ*B Activation to Induce TNF-*α* Production

**Figure 7:**
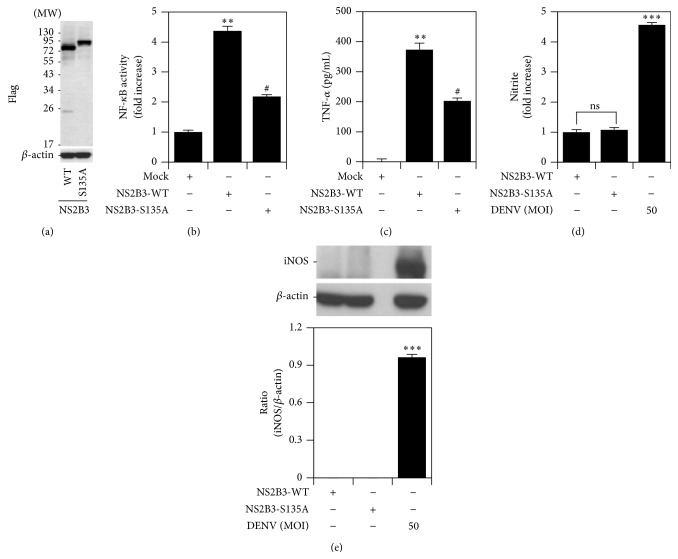
DENV protease NS2B3 also induces NF-*κ*B activation followed specifically by TNF-*α* expression. (a) Western blot analysis showing the expression of NS2B3 in Flag-tagged wild-type (WT) and a mutated NS2B3^S135A^ (*S135A*)-transfected RAW264.7 cells. NF-*κ*B reporter assay (b), ELISA (c), Griess reagent (d), and western blot analysis (e) quantified the activation of NF-*κ*B and the expression of TNF-*α* and iNOS/NO in transfected RAW264.7 cells. ^*∗∗*^ *P* < 0.01 and ^*∗∗∗*^ *P* < 0.001, compared with the mock. ^#^ *P* < 0.05, compared with WT NS2B3. ns: not significant. For western blot results, one set of representative data obtained from three independent experiments is shown. The relative ratio to *β*-actin based on densitometer quantification and analysis using ImageJ software is shown. For all experiments, the quantitative data shown represent the mean ± SD values of three independent experiments.

A recent study showed that the DENV protease NS2B3 activates NF-*κ*B by cleaving I*κ*B-*α* and I*κ*B-*β* [19]. Therefore, to investigate whether NS2B3-activated NF-*κ*B also participates in the induction of TNF-*α*, NO, and iNOS, we overexpressed a wild-type form of NS2B3 (NS2B3-WT) and a protease-dead mutant NS2B3, which has a single point mutation changing serine residue 135 of NS3 to alanine (S135A) that abolished protease activity. Western blotting confirmed that the overexpression of NS2B3-WT and NS2B3-S135A worked efficiently in RAW264.7 cells. Western blotting detected the proform and cleavage form of NS2B3 in NS2B3-WT cells but only one band in NS2B3-S135A cells, demonstrating the loss of NS2B3 autocleavage ability (Figure 7(a)). NF-*κ*B activity was enhanced by overexpressing NS2B3-WT but decreased by overexpressing NS2B3-S135A (Figure 7(b)). Distinct from TLR3 signaling, NS2B3-S135A suppressed TNF-*α* expression (Figure 7(c)) compared with the NS2B3-WT sample. In addition, neither NS2B3-WT nor NS2B3-S135A induced iNOS/NO biosynthesis (Figures 7(d) and 7(e)). These results reveal that forced expression of DENV protease NS2B3 induces NF-*κ*B activation followed by TNF-*α* expression but not iNOS/NO biosynthesis.

## 4. Discussion

**Figure 8:**
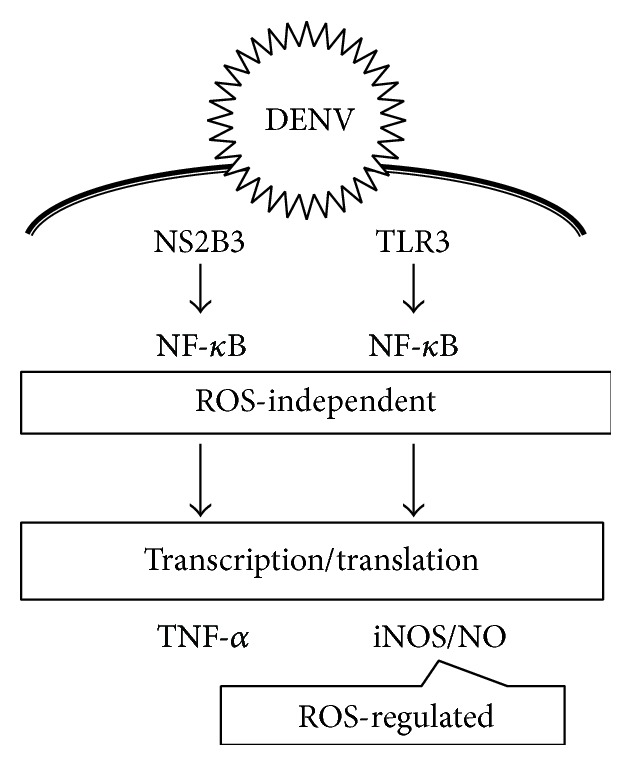
A hypothetical model of the distinct pathways of DENV-induced NF-*κ*B activation followed by TNF-*α* and iNOS/NO expression.

In this study, we identified the distinct pathways of NF-*κ*B activation for regulating iNOS/NO and TNF-*α* expression during DENV infection in RAW264.7 cells. As summarized in Figure 8, the viral protein NS2B3 causes NF-*κ*B activation specifically for TNF-*α* expression and, alternatively, TLR3 mediates NF-*κ*B activation for iNOS/NO biosynthesis. ROS is not involved in NF-*κ*B activation, as demonstrated in DENV-infected RAW264.7 cells. While this is not consistent with previous studies that have shown crosstalk between ROS and NF-*κ*B activation [14], the presence of ROS remains important for the generation of nitrite (NO_2_^−^). We speculate that ROS may directly modulate iNOS activity or indirectly regulate nitrite generation. It may have a limitation on our findings that these results are demonstrated in murine macrophages. In the future, the findings are suggested to be further confirmed in human primary macrophages.

It has generally been suggested that TLRs are required for triggering NF-*κ*B-mediated expression of downstream target genes of proinflammatory mediators during the induction of inflammatory responses in DENV infection. In addition to TLR3, we confirmed an alternative activation of NF-*κ*B caused by viral protein NS2B3. These results show the diverse effects of molecular regulation on NF-*κ*B during DENV infection. In activated macrophages, the activation of NF-*κ*B usually induces inflammatory cytokine TNF-*α* and iNOS/NO generation by controlling the transcriptional activation of the genes [23]. Even under NF-*κ*B activation, the different upstream signaling pathways and coactivators may contribute considerably to inducing specific genes in response to different stimuli. A previous study reported NF-*κ*B-mediated IL-8 expression [24]. The positive transcription elongation factor b, DENV core, and nonstructural protein 5 may also synthetically regulate* IL-8* mRNA transactivation [25, 26]. For DENV-induced expression of TNF-*α* and iNOS in macrophages, other factors involved in the transcriptional activation of* TNF-α* and* iNOS* need further investigation.

During DENV infection, the activation of NF-*κ*B has been widely reported to activate apoptosis in neuroblastoma, hepatoma, and endothelial cells [16, 19, 27], accompanied by chemokine production in endothelial cells [17], IL-8 production in monocytes, epithelial cells, and endothelial cells [24], MHC production in hepatoma cells [28], and type I IFN response in lung epithelial cells [29]. However, the molecular mechanisms underlying the activation of NF-*κ*B caused by DENV infection or viral proteins remain largely undefined. DENV nonstructural protein 1 can increase NF-*κ*B activity in hepatoma cells [30]. Consistent with a study demonstrating that NS2B3 is required for DENV-induced NF-*κ*B activation in endothelial cells [19], we showed an NS2B3-mediated NF-*κ*B activation of TNF-*α* expression in DENV-activated RAW264.7 cells. Interestingly, DENV-infected RAW264.7 cells also underwent apoptosis 48 h after infection (data not shown). For NS2B3-mediated NF-*κ*B activation in endothelial cells [19], the endogenous NF-*κ*B inhibitors I*κ*B*α* and I*κ*B*β* are cleaved by the protease activity of NS2B3. In our study, protease-dead mutant NS2B3 S315A-transfected RAW264.7 cells lost the ability to trigger NF-*κ*B activation and TNF-*α* expression compared to wild-type NS2B3. The NF-*κ*B-promoting effect of downregulating I*κ*B*α* and I*κ*B*β* may need to be confirmed in DENV-infected macrophages.

An increase in TNF-*α* may contribute to the pathogenesis of dengue diseases by modulating cell death and survival, inflammation, and endothelial dysfunction [31]. Although TNF-*α* may not contribute to viral replication directly [32], overproduction of TNF-*α* may further cause NF-*κ*B activation, which has important consequences for NF-*κ*B-driven release of inflammatory cytokines during DENV infection [33]. According to our findings, NS2B3 may be a target for inhibiting DENV-induced TNF-*α* overproduction. In addition to NS2B3, we also showed a TLR3-mediated NF-*κ*B activation of iNOS/NO biosynthesis. The induction of iNOS/NO biosynthesis may contribute not only to inflammatory response but also to initiation of NO-mediated antiviral activity [34]. Regarding the canonical pathway of TLR-3-mediated antiviral response, it is speculated that a TLR3-mediated iNOS/NO induction may be increased for an antiviral purpose in DENV-infected macrophages.

In conclusion, this work identifies two different pathways for NF-*κ*B activation in DENV-infected RAW264.7 macrophages: through the viral protein NS2B3 and through the host pattern recognition receptor TLR3. The viral protein NS2B3-regulated production of TNF-*α* may represent an inflammatory response with a potential immunopathogenic role in DENV infection. In contrast, TLR3-regulated iNOS/NO biosynthesis may be a host response to confer antiviral activity. Obviously, both TNF-*α* and NO may modulate inflammatory activation in DENV-infected macrophages.
